# Knife cut marks inflicted by different blade types and the changes induced by heat: a dimensional and morphological study

**DOI:** 10.1007/s00414-021-02726-5

**Published:** 2021-10-29

**Authors:** Vijarn Vachirawongsakorn, Jonathan Painter, Nicholas Márquez-Grant

**Affiliations:** 1 Department of Forensic Medicine, Faculty of Medicine Siriraj Hospital, Mahidol University, Bangkok, Thailand; 2 Cranfield Forensic Institute, Cranfield University, Defence Academy of the United Kingdom, Shrivenham, UK

**Keywords:** Pre-burning trauma, Sharp force trauma, Skeletal trauma analysis, Forensic anthropology, Cut marks

## Abstract

Detailed information on skeletal trauma analysis of burned bone is important to ascertain the manner and cause of death in forensic casework. This research used three different knife types, one with a non-serrated blade, one a fine-serrated blade, and one a coarse-serrated blade, to inflict trauma to manually macerated *Sus scrofa* ribs (*n* = 240), and these ribs were later exposed to heat. Qualitative and quantitative analyses were conducted using macroscopic and microscopic techniques to assess specific characteristics of the cut marks. Differences in cut mark dimension and morphology of the ribs were investigated. After heat exposure, the cut marks on the rib samples remained recognisable and did not alter considerably. A level of dimensional and morphological preservation was reliant on the cutting action and the features of the knife blade as well as surrounding bone injury. The cut marks inflicted by the non-serrated blade remained recognisable despite exposure to the burning process. However, the cut marks inflicted by the coarse-serrated blade were likely to change significantly when exposed to heat. This study leads to two important results: (1) identification of pre-existing cut marks prior to heat exposure is possible in reconstructed burned bone fragments, and (2) cut marks from different types of knife blades showed dissimilar responses to heat. The outcomes obtained in this study stressed the need to adopt great care with the effects of heat on skeletal trauma analysis.

## Introduction

Sharp force injury is produced by a tool with a sharp edge or pointed object [1]. Death from sharp force injury is a frequent cause of homicidal death, particularly in countries with strict firearm-controlled legislation and policy [2–7]. The most commonly utilised and extensively researched sharp weapon is a knife. This study focuses on a knife being used in a back-and-forth cutting action. If the knife contacts and cuts into the bone, it is most likely that the impact will result in a relatively shallow V-shaped kerf or groove with a clear apex [8]. Yet, many morphological traits can be further defined to describe the features within a cut mark. Detailed analysis of cut marks is influential in interpreting the tool class and can enable a match with the weapon found in medicolegal investigations [7–15].

The macroscopic and microscopic analysis of cut marks found in skeletal remains, along with their interpretation, remains challenging. Research has revealed how several features of cut marks in the bone (termed class characteristics) can be successfully associated with different weapon types; furthermore, individual characteristics within the cut mark can link to a specific tool [16–19]. This study focuses on the class characteristics, and within the research in this area, different bone elements have been employed, both human and non-human, and there seems to be a high correlation between cut mark morphology and tool characteristics [16, 20]. For example, a cut mark made with a serrated blade is likely to record characteristics of a part of the serrated morphology in the floor and walls of the bone defect [21–25].

Heat and flame have effects on skeletal remains resulting in substantial modification of the microstructure. The process of burning the bone is essentially a process of dehydration, elimination of organic matter, and the reorganisation of inorganic substances [26, 27]. The heat-exposed bone is defined as calcined when all organic and water content has been removed [28]. These changes lead to a heat-induced change in macroscopic and microscopic appearance, colour, size, and weight [29, 30]. Specifically, the bone is subjected to the corresponding alterations such as deformation, dimensional change, fragmentation, colour change, and heat-induced fracture [26–28, 30, 31]. Defined by location and direction of fracture propagation, heat-induced fractures can be categorised as longitudinal, transverse, curved transverse, delamination, and patina [32]. Heat-induced fractures and fragmentation can be recognisable, but they may be confused also with skeletal perimortem trauma because their features mimic or obscure antecedent traumatic characteristics [32–34].

Generally, the majority of fire-related deaths are accidental; however, some are deliberately set to obliterate homicidal evidence, including identification, cause and manner of death, and other forensic evidence. Despite the fact that it is difficult for human remains to be completely eliminated by the burning process, fire can profoundly affect the area where there is suspected trauma [32]. Fire modification of the bone can therefore create difficulties for trauma analysis [35]. Recently, studies on the effects of burning on the identification of cut marks have become more prevalent in the forensic literature. Previous studies have shown that cut marks in burned bones remain identifiable and how each mark can be differentiated from heat-induced fractures of the bone [32–34, 36–38]. However, despite surviving the burning process, the heat can modify the characteristics of cut marks in the bone. Various analytical methods have been used to examine the morphology of sharp-inflicted trauma on unburnt and burnt bones. Microscopic methods, such as stereomicroscopy and scanning electron microscopy, are the most commonly employed [27, 32, 33, 35, 36, 38–41].

To that end, the aim of this study is to assess cut marks on non-human ribs using different bladed knives and to then investigate the changes to the cut marks after heating the bones in a furnace. In addition, in subsequent work to be published, further experimentation was undertaken to explore how surface and buried environments modified the initial trauma pattern.

## Materials and methods

To investigate the effect of burning bones on the cut mark morphology, 240 *Sus scrofa* (domestic pig) ribs were cut with three different types of metal knives. This was in addition to 64 ribs used as non-traumatised controls, making it a total of 304 ribs. Domestic pig bones are commonly used in the study of trauma in forensic anthropology [7, 17, 32, 42–44]. These fresh and semi-fleshed pig ribs were collected from a local butcher shop. Only ribs with a fairly consistent size and morphology were selected for the study; hence, the 5th–8th ribs were used in this experiment. The ribs were approximately 25 cm in length and 7 cm in width. The majority of adherent muscle, tendon, and periosteum were removed manually using scissors and forceps ensuring no tool contact with the rib surface. If antemortem bone lesion was identified by visual examination, the rib was excluded from this study. The ribs were frozen when not in use and allowed to thaw in a fridge for 24 h prior to any preparation/experiment.

**Fig. 1:**
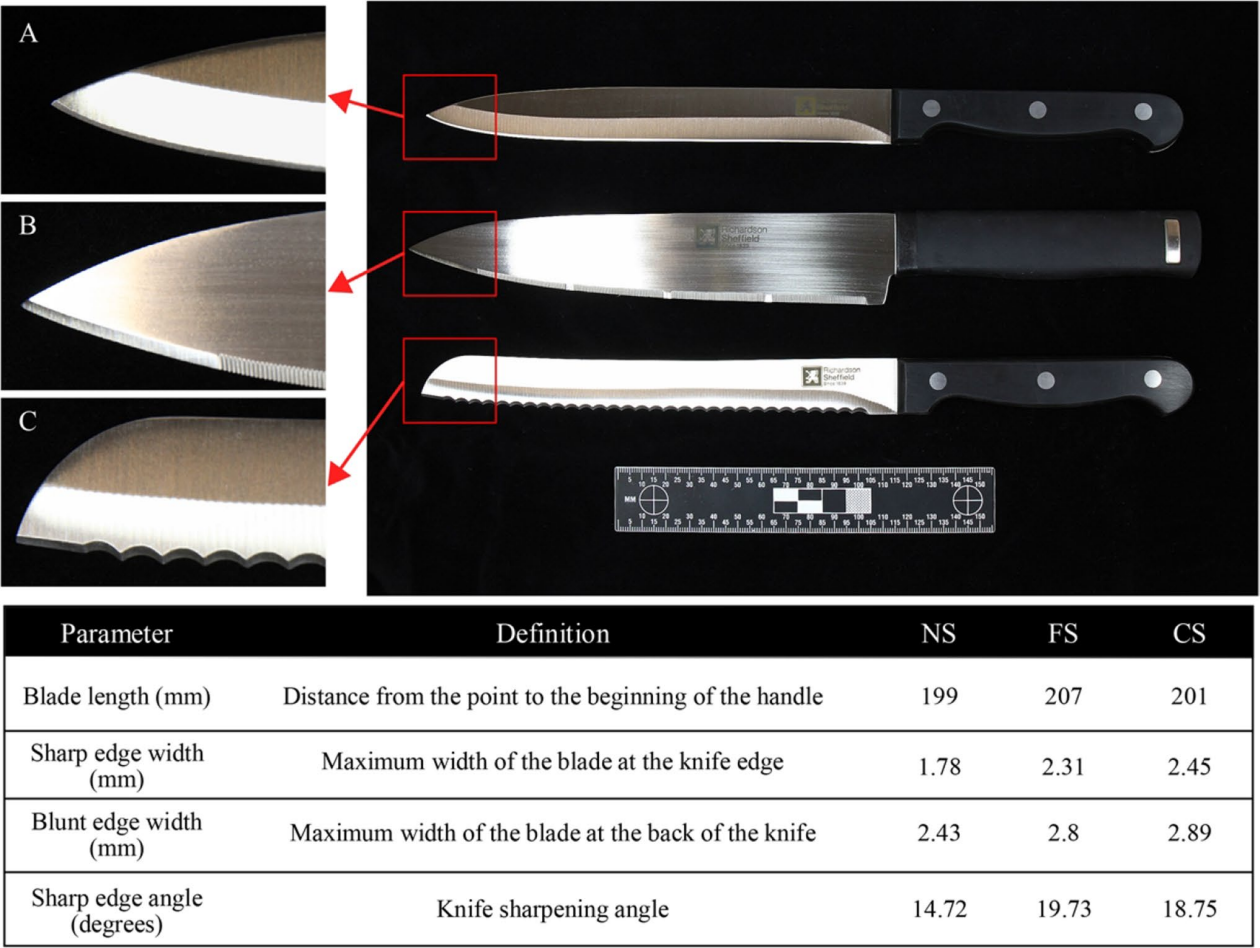
The three knives used for the rib infliction in this study: **A** non-serrated knife, **B** fine-serrated knife, and **C** coarse-serrated knife

Three types of single-edged kitchen knives were purchased for this study, as they are the most common weapons used in sharp force injury [5, 45, 46]. The first one had a blade with a non-serrated edge, the second with a fine-serrated edge, and the last one with a coarse-serrated edge. Figure 1 shows the knives and the measurements of the cutting edge for each knife. The difference in the cutting edge width and angle is readily apparent between the different blade types and would clearly be expected to produce cut marks with different class characteristics. Four knives of each type were purchased with each knife used on 60 ribs before being replaced to minimise the effect of a damaged blade.

### Trauma infliction

The ribs were attached to a standard clamp-on-device to stabilise them during incised events. Three cut marks, which were manually inflicted, were produced with each type of knife on the external surface of the ribs. In order to try and ensure repeatability of knife wounds, one of the authors (VV) was the only person to inflict cut marks in this study maintaining a constant angle and consistency of force. VV is a right-handed, 37-year-old male, 174 cm tall and 68 kg. For each cut mark, the respective knife was moved with a cutting motion perpendicular to the longitudinal axis of the rib. In the procedure, the knife blade was moved with a single back-and-forth motion. The blade was then carefully withdrawn, and each mark was individually labelled and photographed. After examination of the cut mark as detailed below, the samples were shrink-wrapped and kept frozen until defrosted for burning.

### Burning procedure for ribs

A laboratory Carbolite® CWF 110 electric furnace was used for the burning process because it can control the duration and temperature conditions of this process, thus allowing uniformity in methodology and the burning process.

Every burning experiment followed the same procedure. A limestone slab was centrally located inside the furnace chamber to raise the samples to a level in line with the thermocouple so as to ensure the specimens were subjected to the desired temperature. On top of the slab, metal baskets were used for the separation of more than two samples at each burning event. The samples were put inside the cold furnace and heated up at an average of 15°C/min until it reached a peak temperature of 850°C for at least 30 min to achieve complete bone calcination [33, 47]. The furnace was then turned off and allowed to cool down slowly to room temperature to prevent post-burning crack formation from sudden cooling [29].

### Analytical techniques

**Fig. 2:**
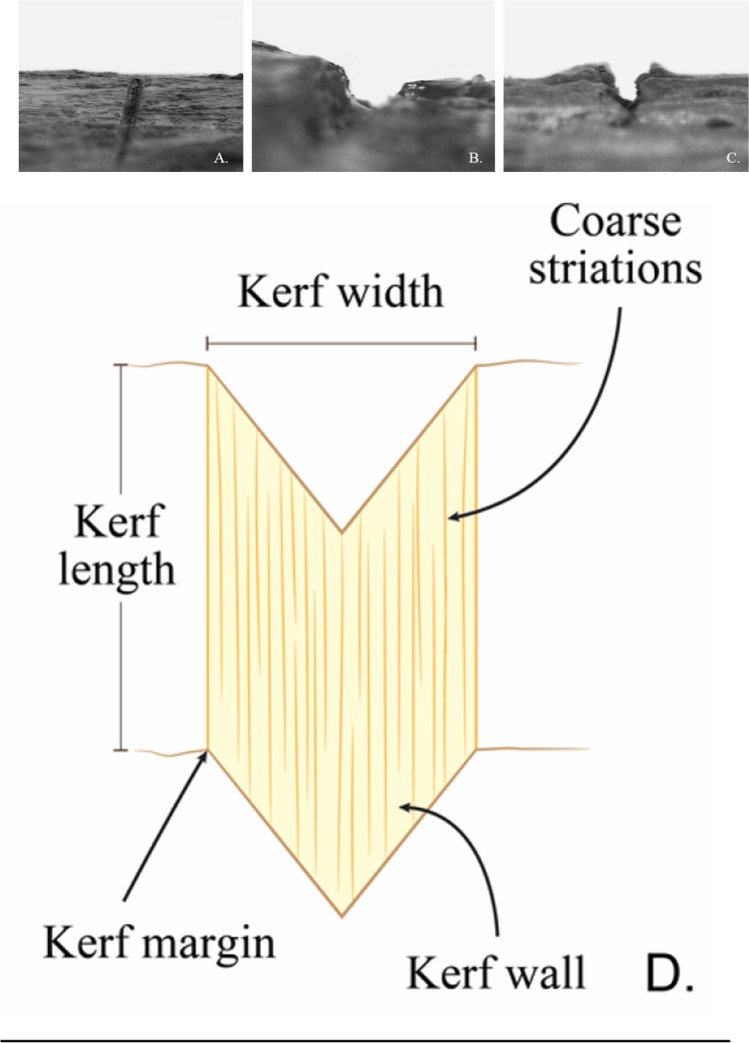
The cross-sectional views of cut marks: **A** narrow shape, **B** U-shaped, **C** V-shaped, and **D** sketch defining the kerf features and measurements

**Fig. 3:**
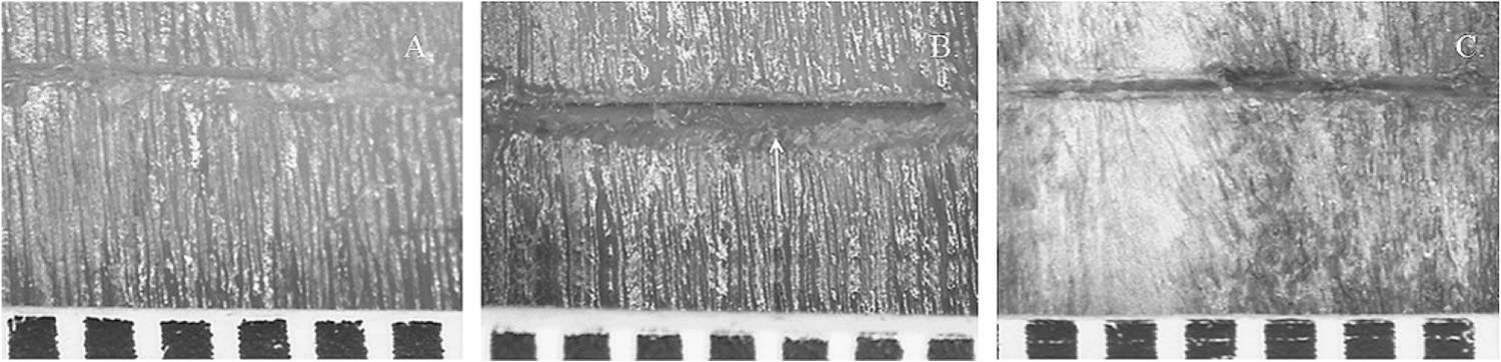
A view of a cut mark inflicted by **A** a non-serrated blade, cutting edge width 1.78 mm; **B** a coarse-serrated blade, cutting edge width 2.45 mm (the white arrow indicates a raised edge); and **C** a fine-serrated blade, cutting edge width 2.31 mm; scale in mm

To evaluate any changes to the trauma morphology due to the effect of heat, each cut mark was characterised before and after burning. The assessment of the trauma used both the naked eye and stereomicroscopic examinations to determine qualitative and quantitative characteristics of the cut mark morphology. These characteristics, detailed in Table 1, include overall morphology, cross-sectional morphology, marginal morphology, and presence or absence of coarse striations [14, 20]. Figure 2 shows example of side-on images of 3 cut marks, depicting the variation in kerf cross-sectional shapes referred to in Table 1, as well as a sketch defining the kerf measurements. It is important to point out that the class characteristic assessment of the cut marks were conducted directly on the ribs, not by assessing casts of the cut marks. The reason for this is that although this paper is analysing cut marks pre- and post-burning, the same samples were used to subsequently study the effects of environmental degradation with time (to be published in a future paper). As the degree of degradation was unknown, it was considered possible that repeated casting of the cut marks could compromise the study of the taphonomic alterations or morphological changes. Hence, the direct assessment was preferred; while acknowledging that casting has been shown to be essential for the visualisation of very fine striations [18, 48], the other class characteristics should not be compromised. Therefore, in this paper, striations visible in the cut marks are mainly coarse striations. Bone surface modifications were also examined to evaluate overall heat-related changes. Quantitative measurements include the length and width of the marks. The maximum length measurement was directly determined using a digital sliding calliper. For obtaining the maximum width, each cut mark was examined using an Olympus SZX10 stereomicroscope with an attached XIMEA xiQ digital camera and software (V.3.18.02), and images were acquired of areas of interest (Fig. 3). Measurements were made using ImageJ (Version 1.51g, National Institute of Health, USA), calibrated using a scale, with the measurement conducted perpendicularly at the broadest point of the kerf mark. Each kerf dimension was repeatedly measured three times to obtain an average value.

**Table 1:** Morphological variables and analytical processes; E, naked eye examination; M, stereomicroscopic examination

Variables	Method		Definition	Findings and their definitions
	E	M		
**Kerf length**	X		Maximum distance between the starting and ending point of a kerf	-
**Kerf width**		X	Maximum distance between the outermost margins of a kerf	-
**Kerf shape**	X	X	Overall top-view shape of a kerf	Linear: Narrow and parallel kerf margins and walls Elliptical: Two inward-angled kerf margins that end at their tips with the broadest distance at the middle of the kerf Rectangular: Parallel kerf margins and walls that end at their small U-shaped tips Irregular: Irregularity of kerf morphology that cannot be categorised into any types of kerf shape
**Kerf margin**	X	X	Marginal morphology of a kerf	Smooth: A regular, smooth, and flat kerf margins Raised: An uneven, lateral raised margin of a kerf that attaches to the bone
**Cross-sectional view**		X	Cross-sectional shape of a kerf	V-shaped: Two inward-angled kerf walls that end at the floor with the widest distance at the margin and narrower when the kerf walls descend to their floor U-shaped: Two parallel walls that are connected by a curved floor with an equal distance between the walls Narrow: Two parallel walls with a particularly narrow distance between the kerf walls and margins
**Coarse striations**		X	Parallel striations on a kerf wall	Present, absent

### Statistical analysis

The data was analysed using IBM SPSS Statistics Version 18.0 (IBM Corp.). A statistical significance between different types of inflicted knives was investigated using a Student’s t-test, after checking for normal distribution. Also, the morphological differences of cut marks inflicted by each type of knife were analysed using Chi-square test and Fisher’s exact test.

## Results

### Pre-burned bones

All the cut marks (*n*=720) were initially assessed and characterised, with the most common features of each knife type used in this study given in Table 2. An example of a cut mark from each of the blade types is shown in Figure 2.

**Table 2:** Diagnostic cut mark characteristics for each knife class (each *n* = 240) with the proportion given as %

Kerf feature	Non-serrated blade	Coarse-serrated blade	Fine-serrated blade
Kerf shape	Linear (100%)	Elliptical (80%)	Elliptical (92.5%)
Cross-section	Narrow (100%)	V-shaped (55%) or U-shaped (45%)	V-shaped (95%)
Kerf margin	Smooth edge (100%)	Raised edge (58.3%)	Smooth edge (56.7%)
Kerf coarse striations	Absence (100%)	Striations (60%)	Striations (67%)

**Fig. 4:**
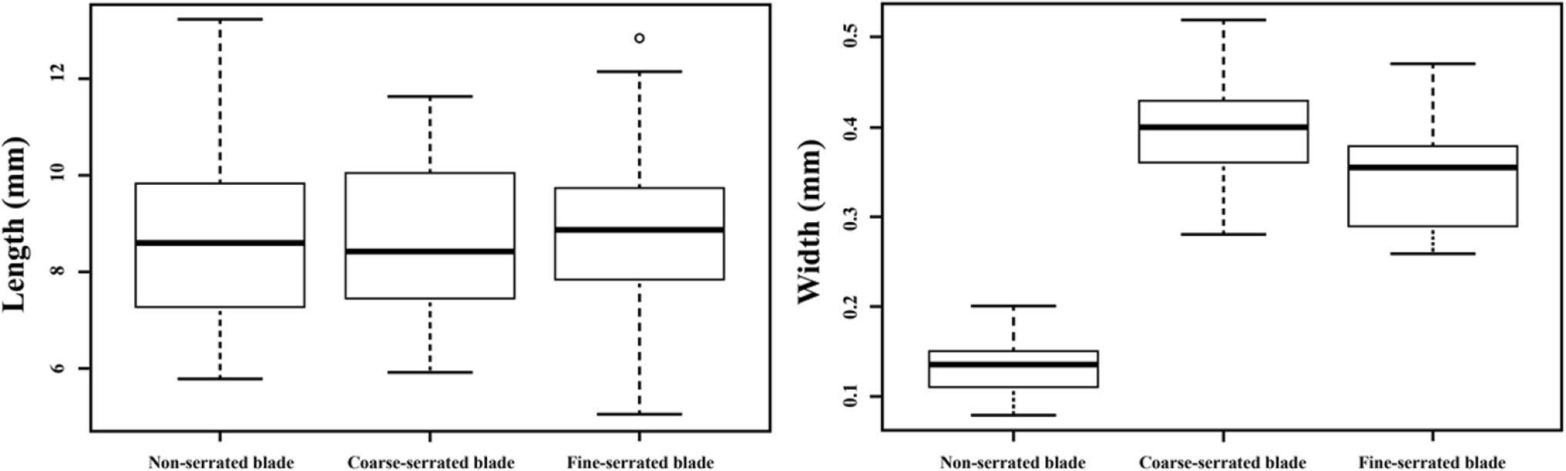
Maximum and minimum range of length (left) and width (right) produced by a non-serrated knife, a coarse-serrated knife, and a fine-serrated knife; each *n* = 240

Boxplots comparing the kerf length and width of the different types of knife blade are shown in Figure 4. As shown, a wide overlap of kerf lengths produced by the different types of knife blade was observed, as would be expected as the cutting action was intended to be identical. However, the kerf width shows clear differences between the serrated and non-serrated blades used in this study.

The kerf morphology for all the cut marks with the different types of the knife blade is summarised in Table 4. To further examine differences between blade types, statistical analyses of the characterised cut mark morphological characteristics were carried out (Table 3). All three types of knife blade produced cut marks that showed significant statistical differences in their maximum width and kerf shape, whereas there was no statistical significance of the maximum length. Cut marks produced by the non-serrated blade knife were statistically different in their kerf maximum width and morphology from those produced by the coarse-serrated and fine-serrated blade knives.

**Table 3:** Statistical tests of pre-exposure kerf dimension and morphology between cut mark inflicted by non-serrated blade (NS), coarse-serrated blade (CS), and fine-serrated blade (FS) (*** Significant statistical difference)

Kerf dimension or morphology	Statistical values		
	NS and CS	NS and FS	CS and FS
Maximum length	*t* = − 0.124, *df* = 118, *p* = 0.902	*t* = 0.012, *df* = 118, *p* = 0.99	*t* = 0.135, *df* = 118, p = 0.89
Maximum width	*t* = 21.57, *df* = 118, *p* < 0.001***	*t* = 17.82, *df* = 118, *p* < 0.001***	*t* = − 3.512, *df* = 118, *p* < 0.001***
Kerf shape	Fisher’s test: *p* < 0.001***	Fisher’s test: *p* < 0.001***	Fisher’s test: *p* = 0.024***
Cross-section shape	Fisher’s test: *p* < 0.001***	Fisher’s test: *p* < 0.001***	Fisher’s test: *p* = 0.67
Kerf margin	Fisher’s test: *p* < 0.001***	Fisher’s test: *p* = 0.0046***	Fisher’s test: *p* = 0.58
Striation	Fisher’s test: *p* < 0.001***	Fisher’s test: *p* < 0.001***	*X*^2^ = 0.0718, *df* = 1, *p* = 0.789

### Post-burned bones

**Fig. 5:**
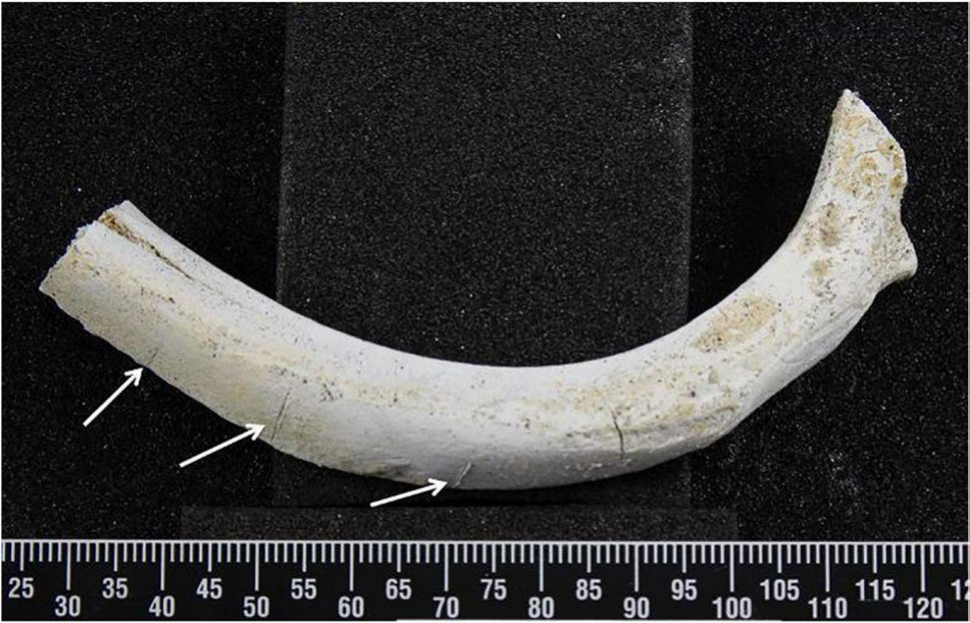
A rib after burning; the white arrows indicate cut marks; scale in mm

After the burning process, all samples were completely calcined. The rib samples were burned uniformly, yet a range of bluish-grey discoloration was observed on parts of some of the ribs. For each bone burnt, the ribs largely maintained structural integrity although a few fragments of the burned bone were retrieved from the bottom of the furnace. Despite the burned ribs becoming very fragile and having a chalky appearance, all the cut marks remained identifiable (Fig. 5).

**Fig. 6:**
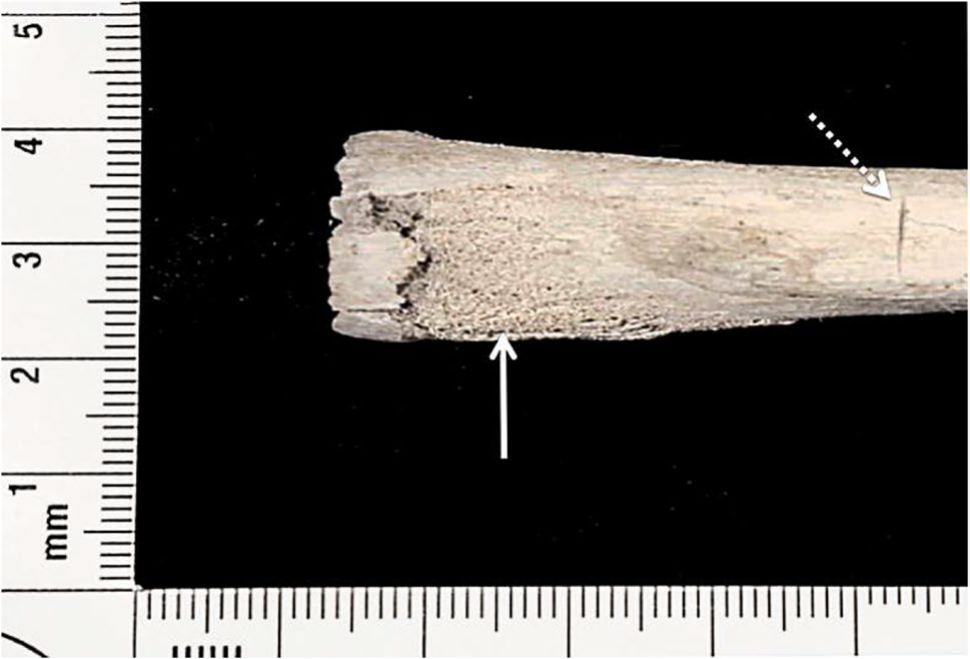
Delamination (the white arrow) of a calcined rib; the dotted arrow identifies a cut mark

Visual observation of the ribs revealed that there were heat-induced fractures identified in almost all (97.5%) of the traumatised and non-traumatised samples. Delamination was the most common heat-induced fracture found in the current study, appearing in 97.5% of rib samples (Figure 6). Longitudinal and straight transverse heat-induced fractures were found in 70% and 32.5% of sample findings, respectively. In addition, 30% of the ribs were found to have warped as a result of heat exposure, although all ribs were unfleshed. There was no statistical difference in the heat-induced fracture characteristics of ribs either with or without cut marks (*p*=0.43).

**Fig. 7:**
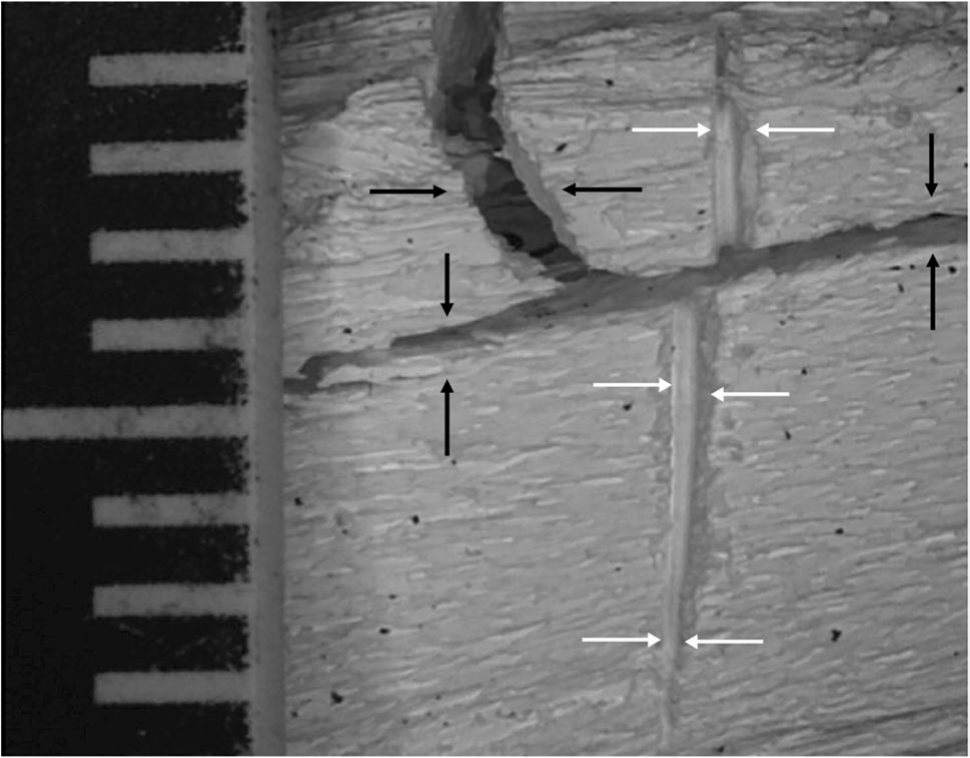
A cut mark transected by a heat-induced fracture; white arrows indicate the cut mark after thermal influence; black arrows indicate the heat-induced cracks; scale in mm

**Fig. 8:**
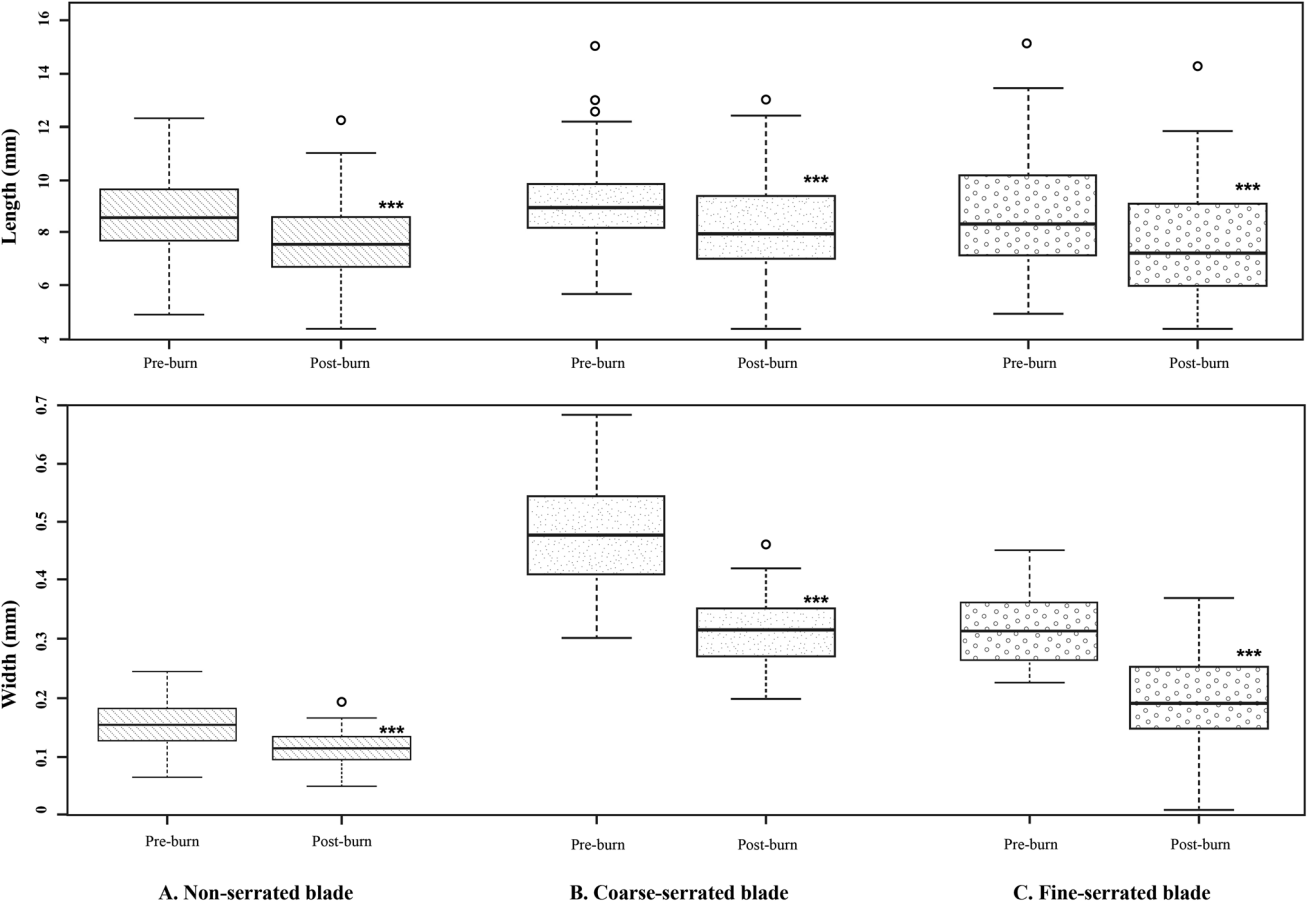
Boxplots illustrating a distribution of kerf length and width data (three asterisks indicate statistical significance of the same sample between pre- and post-burn data)

All the cut marks were visible after burning, even in areas where heat-induced fractures intersected the trauma marks; Figure 7 is one such example. For those cut marks that were unaffected by such intersections, the changes to the kerf length and width for all the undamaged cut marks are illustrated in boxplots in Figure 8. Significant decreases of post-cremated length and width (*p*<0.05) were observed, with a decrease in kerf length of between 10.8 and 17.6% and width of between 28.5 and 34.9%. Measurements of the cut marks affected by fragmentation damage or heat-induced fracture were not undertaken, but these formed only a small proportion of the total cut marks (5.8%).

Morphological characteristics of all the cut marks before and after burning are shown in Table 4. Statistical analysis of these characteristics was undertaken to enable identification of those features which change due to the burning and whether this varied with blade type. A significant morphological change (*p*<0.05) is seen in kerf shape and kerf margin of cut marks produced from a coarse-serrated blade knife. Concisely, 26.6% of marks with an elliptical kerf shape changed to a rectangular and irregular shape, and 26.4% with a raised kerf margin became smoother margin after burning, whereas cut marks inflicted by a fine-serrated blade knife changed significantly (*p*<0.05) only in kerf shape morphology.

**Table 4:**
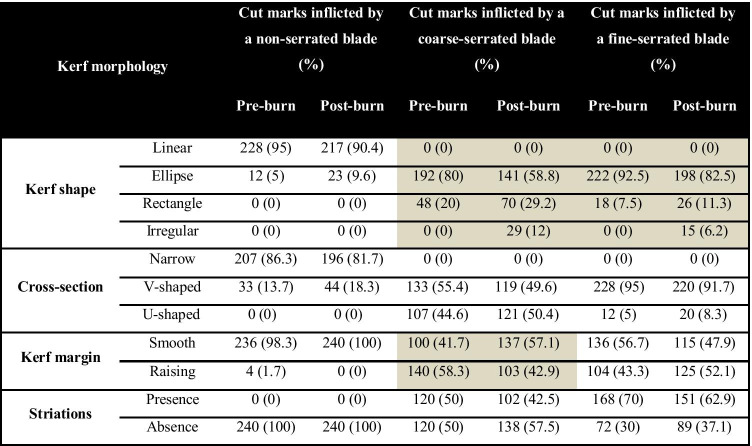
Summary of frequency data of kerf morphology between pre-burnt and post-burnt samples; shading demonstrates statistical significance

**Fig. 9:**
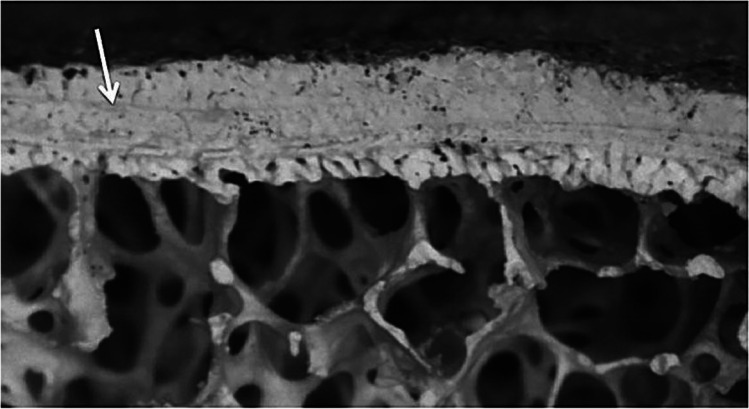
Stereomicroscopic examination of kerf coarse striations in a cut mark (the white arrow) after the bone was burnt

**Fig. 10:**
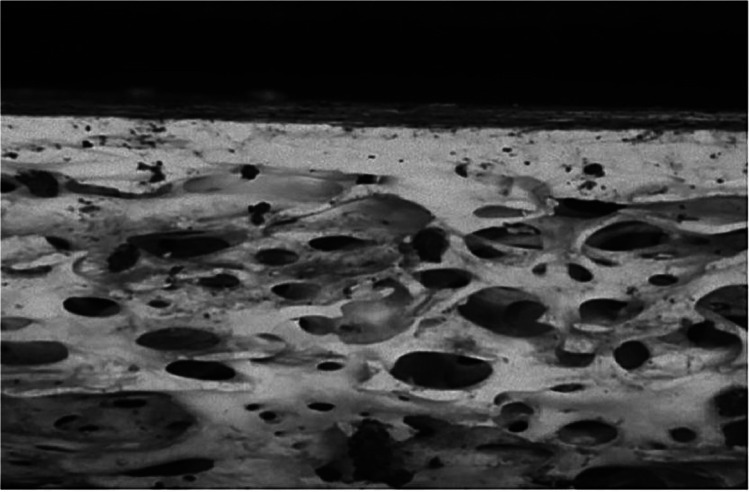
Microscopic examination of heat-induced fracture

Almost all kerf coarse striations could be recognised after the burning event, meaning that whereas fire affects some of the characteristics of cut marks, it does not necessarily destroy them all (Fig. 9). The edges of heat-induced fractures generally had noticeably smoother surfaces than the cut mark walls, irrespective of blade type (Fig. 10). The heat fracture surfaces were smooth in 82.2% of the longitudinal and 64.5% of the straight traverse fractures.

## Discussion

### Pre-burning

In this study, 720 cut marks, on 240 defleshed pig ribs, using 3 different types of knifes, were made using manual infliction by a single human. The choice of kitchen knives in this study were those commonly found to have been used in violent cases [2, 4, 5, 45]. Newly purchased kitchen knives were used to minimise the chance of blade defects imparting additional features in the cut mark [10, 21, 49, 50]. Despite trying to ensure that a consistent force and angle were applied each time, a variety of sizes and shapes of cut marks were observed even when the same weapon was used. This is an inevitable consequence of manual infliction, but also the natural variation between ribs, resulting in a variation in the blade/rib interaction. These findings are in line with previous studies [21, 49]. Further studies using a cutting rig with defined force and angles may be conducted to reduce the human variations.

It is important to note that a variety of cut mark dimensions and morphological characteristics are expected when using different knife blade types; the difference in the blades’ cutting edge characteristics is reported earlier in Figure 1; cut marks created and examined in this study have characteristics which are in general agreement with previous studies [8, 51–53]. The profile of the cut marks inflicted by a non-serrated blade was found to be linear-shaped, a narrow cross-section, and with no striations which are indications of this knife type. Conversely, a coarse-serrated blade normally produces an elliptical V- or U-shaped cross-sectional cut mark with a raised or smooth margin and striated wall. A fine-serrated blade knife produces an elliptical shape with a V-shaped cross-section and smooth or raised margins with a striated kerf wall. The exact relationship between the knife and cut mark cross-section and dimensions needs more detailed investigation in future studies, with profiles and measurements of the cross-sections of the blade edges correlated to the cut marks.

#### Kerf dimension

Previous studies have shown that a cut mark inflicted by a non-serrated blade tends to be narrower than those made by a serrated blade [18, 50, 53]. The results of this study were consistent and showed a significant statistical distribution (p<0.001) of the kerf width differences from non-serrated, coarse-serrated, and fine-serrated blades (Table 3). It was observed that the order of increasing cut mark width was the same as the cutting edge width of the blade. In relation to the cutting edge angle of the blades, there were differences. While the non-serrated blade had the smallest blade edge angle and the narrowest cut mark, the cut mark from the coarse blade knife was wider than that for the fine-serrated blade. This is despite the cutting edge angle of the fine-serrated blade being larger than that for the coarse-serrated blade. This indicates that the larger serrations and wider blade of the coarse-serrated knife influenced the cut mark width beyond that of the cutting edge angle. It is however noted that, due to manual infliction used in this and other studies, variations in the force and angle of the blade could produce wider cut marks due to the angle of impact and force applied [54, 55]. Therefore, further study still needs to be conducted with different types of knife blade to determine the kerf width with weapon type. In contrast, the kerf lengths from the different blade types were found to be similar, with variations likely due to small fluctuations in the knife force/angle and/or rib shape. Thus, no association between the kerf length and knife blade type has been proven in this study in contrast to Humphrey et al (2017) [55], indicating further research is needed.

#### Kerf morphology

Several characteristics of kerf shape were described comprising linear, elliptical, rectangular, and irregular shapes. Statistical significant differences (*p*<0.05) between the different knife types and kerf shapes were observed (Table 3) indicating that the kerf shape is specifically related to the size and morphology of the blade (blade characteristics are given in Figure 1). Briefly, all cut marks produced by a non-serrated blade displayed a linear and narrow kerf shape due to the narrow knife edge, whereas the shape of cut marks inflicted by coarse-serrated and fine-serrated blades was predominantly elliptical, 80% and 92.5% of marks, respectively. An elliptical shape arises from the geometry of a V-shaped blade and a cylindrical surface. However, some variation is observed. A large serrated blade produces mostly elliptical or rectangular shapes, the latter due to the deeper penetration into the bone. A fine-serrated blade usually produces a cut mark with an elliptical shape because its small serration makes good contact when cutting through the bone surface.

Normally, a V shape is the most common characteristic of the cross-sectional shape observed in knife cut marks [14, 56]. Lewis (2008) [57] advised using this feature to distinguish knife type. Nonetheless, Cerutti et al. (2016) [52] stated that this feature could not be used to differentiate between different types of knife blade because a V-shaped feature can be produced from any type of a knife blade. This study showed a variety of cross-sectional kerf mark shapes, but there were relationships between the cross-sectional shape and the blade type. Specifically, some cross-sectional shapes can be found more frequently with a specific type of knife blade. Most of the cut marks made by non-serrated blade knives displayed narrow cross-sections, with the remaining 13.7% being V-shaped. Around 55% of cut marks inflicted by a coarse-serrated blade had a V-shaped cross-section, while the remaining had a U-shaped cross-section. In addition, 95% of cut marks inflicted by fine-serrated blade produced V-shaped marks, with the remaining having U-shaped cross-sections. These results support a trend that the definition of the V shape varies depending on the size and shape of the blade [8, 13, 18, 21, 49, 54–57]. A U-shaped cross-sectional shape can be formed by a saw and other types of broad blade weapons such as a stone tool [10, 13, 56]. A coarse-serrated blade used in this study produced a U-shaped cross-section in 45% of samples (55% V-shaped), a much greater variation in cross-sectional shape than the other blade types. This finding can be explained by the cutting mechanism of this blade type, which is like a handsaw. When a coarse-serrated blade cuts bone, it can cut smoothly, skip over the bone surface, and/or change in cutting direction. This variation in the blade/bone interaction then explains the greater variation in the cross-sectional shape of cut marks inflicted by this blade type. For all three knife types, the degree to which the variations in kerf shape result solely from differences in the angle and/or force of the blade (inevitable with the manual infliction) during the cutting action is unclear, but it should be noted that knife impact differences could be a contributing factor.

A raised kerf margin is defined as having ragged and deformed margins along cut mark edges. In this study, the kerf margin showed a correlation to the cutting edge feature of the inflicted blade. The cut marks made by a coarse-serrated blade had the most frequent incidence of raised margin (58.3%), followed by those inflicted by a fine-serrated blade (43.3%). Conversely, the cut marks inflicted by a non-serrated blade showed only smooth margins. These findings are consistent with previous works suggesting cut marks with raised margins are made by serrated knives [7, 14, 20]. Tennick (2012) [14] stated that a raised margin tends to be the result of the interaction between a blade edge and a bone surface. Chattering, scraping, and skipping are commonly found in the case of cutting with serrated blade teeth, and it is these mechanisms which result in the raised margin. Likewise, the number and pattern of serrated blade teeth may play a role in the likelihood of these cutting artefacts. A fine-serrated blade has smaller and compact teeth providing better grip on a bone surface, and therefore a fine-serrated blade is more likely to create a smooth and regular kerf margin. Conversely, a coarse-serrated blade with fewer and larger teeth provides a poor grip on a bone surface resulting in greater skip and chatter.

In this study, an increased percentage with kerf striations in the cut marks inflicted by fine-serrated (67%) and coarse-serrated (60%) blades and the lack of this characteristic in the cut marks inflicted by a non-serrated blade indicate that kerf striations are useful to distinguish knife class, as found by others [20, 50]. In addition, the morphology of the striations varied with the two different types of serrated knives, indicating that the blade/teeth size and thickness influence the striations, as also found in other studies [14, 53]. A fine-serrated blade usually produces smaller and more delicate kerf striations because it interacts closely with a bone surface. However, in this study, not all cut marks from serrated blades contained visible kerf striations, particularly those produced by the fine striated blade; hence, the lack of striations cannot be used to rule out a serrated blade. Although this may be a result of the cutting, it is highly likely that a major factor is due to the difficulty of visualising the fine detail on the partially translucent/light coloured bone as reported by others, supporting the need to use casting/oblique lighting to enhance surface topography visualisation [48, 58]. As previously described, the decision was made not to cast the cut marks as the fine striations were only a small portion of a much larger study into the persistence of cut mark characteristics.

### Post-burning

For forensic investigations, it is important to be able to detect a traumatic lesion on a burned bone and attempt to classify the type of weapon used. Previous literature showed that sharp force trauma on a bone could survive the bone being burned [26, 32–34, 38, 59]. It is highlighted that morphological changes to the trauma could occur because of the burning; hence, further research is necessary [30, 32–36]. Similarly, all the cut marks in this study were grossly identifiable after burning, despite the bone sample fracturing, with a small number of fractures intersecting cut marks.

#### Kerf dimension

A skeletal element is subjected to an intense loss of water and organic matter during the burning process. Theoretically, these should lead to a marked decrease in cut mark dimensions from a combination of collagen and moisture loss as well as hydroxyapatite recrystallisation [26, 28, 31]. Previous literature showed that heat-induced bone shrinkage has an effect on all bone dimensions especially at high temperature [26, 27, 30, 31, 59–63]. A burned bone starts to shrink at 200°C, but a temperature of around 800°C is a critical temperature at which the extent of heat-induced dimensional changes increases significantly [31, 39, 64].

All samples in this study were burned at 850°C for 30 min. A statistically significant decrease in the kerf length around 10.8–17.6% and the kerf width of 28.5–34.9% compared with the original dimension was observed. Consequently, cut mark dimensional changes are non-uniform and have a directional dependency. The alignment of collagen fibres may play an important role to explain this characteristic. In this study, cut marks were perpendicular to the lengthwise direction of collagen fibres which are aligned along the shaft of the ribs [64–66]. As a result, degradation and shrinkage of collagen fibres from the burning process, contracting the length of the rib, thereby reduced the kerf width more than the kerf length. In addition, warping deformation may influence the kerf dimensions by either pulling or pushing the cut mark walls into the other one [39].

The use of juvenile bone in this study may have affected the degree of heat-induced dimensional changes. If collagen changes are associated with burned bone’s dimensional changes, age should be an important factor too because the proportion of collagen fibre changes during life [67, 68]. Compared to an adult bone, the juvenile skeleton has a higher collagen and lower mineral content. Therefore, upon burning a greater contraction in the direction aligned with the collagen fibres could be expected compared with that in an adult bone. Hence, the age of the individual, as well as the alignment of cut marks with the collagen fibre direction, is likely to affect the cut mark dimensional change after the burning process. This has been reported by some but warrants further investigation [31, 36].

#### Kerf morphology

In this study, heat-induced fractures intersected and therefore damaged a minority of cut marks, but most of the cut marks remained clearly defined after the burning process. However, some heat-induced morphological changes were statistically significant. After the bone samples were recovered from the furnace, ellipse-shaped cut marks from coarse-serrated and fine-serrated blades changed to either a rectangular or irregular shape. In addition, raised margins of cut marks inflicted by a coarse-serrated blade are transformed into smooth margins (Table 4).

The burning-induced changes in the kerf shape of cut marks inflicted by coarse-serrated and fine-serrated blades, as opposed to the stable cut mark from a non-serrated blade, are likely due to the variation in the cutting edge/bone interaction. The teeth on the blade of coarse-serrated and fine-serrated knives cut and chatter over the bone surface, therefore imparting greater mechanical trauma localised around, as well as finer detail within, the cut mark. Thus, the raised kerf margins, seen with the coarse-serrated blade, would be such damaged bone and would be more susceptible to alteration during a burning event. In this study, the raised kerf margins were commonly absent after the burning process although deformed, blackened, and eroded margins were also observed.

Almost all kerf striations could be recognised after the burning event, meaning that whereas fire affects some cut mark characteristics, it does not necessarily destroy them all. The definition of the kerf striations depends not only on the morphological characteristics of the knife but also the ability of the bone sample to receive the cut mark [40, 41]. In comparison with the raised kerf margin, formed from displaced and damaged bone and therefore susceptible to heat effects, this suggests that the more robust striations are not formed in a similar manner, instead are surface topography resulting from the cutting action of the serrated knife blade with the bone.

A distinction between a skeletal traumatic lesion and a heat-induced fracture has been identified in this study. In addition, it was seen that cut marks did not act as nucleation sites or crack paths for fracture hence had a good chance of surviving and being observable post burning. Visually, all cut marks could be recognised and identified by their linear, narrow features with a smooth or raised margin. In contrast heat-induced damages had well-defined borders and a random crack path. Microscopically, burning of the bones resulted in the complete or partial erosion of raised kerf margins associated with cut marks, although fine detail within the marks such as striations was unaltered (Fig. 8), whereas smooth and clean borders were observed in heat-induced fractures (Fig. 9). These findings correspond with advice by Pope and Smith (2004) [34] who stipulate using microscopic analysis of defect borders to distinguish between heat-induced damage and a traumatic fracture. They recommended that reconstruction of a suspected lesion should be first carried out, and this should be followed by microscopic examination [32–34]. Generally, the bone lesions from heat-induced damages and perimortem fractures are the outcomes of the type of loading force and the property of skeletal tissue involved [28, 32, 34, 59, 69]. A heat-induced fracture formed in a brittle bone element does not have as good energy-absorbing property as a normal bone. Therefore, it is dissimilar to a traumatic fracture formed in ductile, fresh bone material. Herrmann and Bennett (1999) [32] summarised that a mechanism of the heat-induced fracture in a burned bone is very similar to a fracture from a high-velocity injury such as a gunshot wound.

## Conclusion

This study has investigated the change to cut mark characteristics following burning. Three different knife types were used to inflict 240 cut marks on pig ribs (720 in total), with the cuts perpendicular to the length of the rib (and therefore the direction of the collagen). The cut marks were first microscopically characterised, burnt in a furnace at 850 °C for 30 min, and then characterised again. As has been found by other researchers, some of the characteristics of fresh cut marks are affected by the type of knife blade. It was seen that although burning the ribs often induced fracturing of the bone, in only 5.8% of cases did the crack intersect a cut mark, there was thus minimal detrimental effect on the ability to fully analyse cut marks. It is also clear that the cut marks did not directly contribute to either the fracture path initiation or propagation direction. Most of the time, the cut marks were identifiable and macroscopically underwent limited alteration. However, microscopically some changes were noted, and those changes varied depending upon blade type, blade/bone interaction, and potentially the orientation of the cut mark with the direction of collagen fibres in the bone. For the latter point, the greater reduction in cut mark width than in the length is attributed to the perpendicular alignment between the cut marks and collagen fibres. This reduction in cut mark dimensions is independent of blade type, unlike the other heat-induced changes. Serrated blades resulted in striations in the kerf wall, which did survive burning unaltered, whereas a raised kerf margin underwent at least partial and sometimes complete erosion during burning. The raised kerf margin was particularly associated with the coarse-serrated blade. The least variation was observed in cut marks inflicted using a non-serrated blade where only a small proportion underwent a shape change, that is, the cut marks retained the characteristics of a non-serrated blade. In this study, the goal was to assess the characteristics and changes arising from burning; hence, the type of knife used was always known when assessing the cut marks. Further work would necessitate the use of blind tests to study the ability to assign cut marks to knife type.

Finally, whereas the cut mark identification and analysis was achievable on burnt bone, the heat fracturing and general fragility of the specimens did complicate the analysis. Therefore, this study further stresses the need to adopt great care when analysing heat-affected skeletal trauma.

## Data Availability

Not applicable.
